# PP2A: A Promising Biomarker and Therapeutic Target in Endometrial Cancer

**DOI:** 10.3389/fonc.2019.00462

**Published:** 2019-06-04

**Authors:** Michiel Remmerie, Veerle Janssens

**Affiliations:** Laboratory of Protein Phosphorylation and Proteomics, Department of Cellular and Molecular Medicine, KU Leuven, Leuven, Belgium

**Keywords:** phosphatase targeted therapy, PPP2R1A, endometrial cancer, type II endometrial carcinoma, serous endometrial carcinoma, CIP2A, PME-1, PP2A activating drug

## Abstract

Over the last decade, the use of targeted therapies has immensely increased in the treatment of cancer. However, treatment for endometrial carcinomas (ECs) has lagged behind, although potential molecular markers have been identified. This is particularly problematic for the type II ECs, since these aggressive tumors are usually not responsive toward the current standard therapies. Therefore, type II ECs are responsible for most EC-related deaths, indicating the need for new treatment options. Interestingly, molecular analyses of type II ECs have uncovered frequent genetic alterations (up to 40%) in *PPP2R1A*, encoding the Aα subunit of the tumor suppressive heterotrimeric protein phosphatase type 2A (PP2A). *PPP2R1A* mutations were also reported in type I ECs and other common gynecologic cancers, albeit at much lower frequencies (0–7%). Nevertheless, PP2A inactivation in the latter cancer types is common via other mechanisms, in particular by increased expression of Cancerous Inhibitor of PP2A (CIP2A) and PP2A Methylesterase-1 (PME-1) proteins. In this review, we discuss the therapeutic potential of direct and indirect PP2A targeting compounds, possibly in combination with other anti-cancer drugs, in EC. Furthermore, we investigate the potential of the PP2A status as a predictive and/or prognostic marker for type I and II ECs.

## Introduction

Treatment options for cancer have advanced immensely throughout history, with mainly one goal: to specifically target the tumor with as little harm as possible for the patient (1–4). During the last decade, molecular characterization of many tumors has brought cancer research another step closer toward this goal (5), providing the keys to unlock the door toward personalized medicine. However, treatment for endometrial cancer seems to lag behind, although potential markers for this disease have been identified and successful precedents using such markers for targeted therapy have been set in other cancers (e.g., lung cancer, chronic myeloid leukemia, breast cancer, melanoma) (6–13).

In this review, we will discuss the potential of the tumor suppressive protein phosphatase type 2A (PP2A) as a new biomarker and therapeutic target for both type I and type II endometrial carcinomas (ECs). We will mainly focus on the potential predictive and prognostic value of *PPP2R1A*, encoding the Aα subunit of PP2A, which is mutated in up to 40% of type II ECs, while largely being unaffected in type I ECs and other common gynecologic cancers. In the latter cancer types, PP2A dysfunction commonly occurs, however, by other mechanisms of inactivation, stressing the importance of functional PP2A for preventing tumor development and/or progression. Overall, we propose that current, unsatisfactory (type II) EC treatments could be largely improved by taking the PP2A status of the tumor into account since it could be a potential useful indicator for prognosis and therapy response.

## Why Targeted Therapies Should be the Focus in Type II EC

To date, the standard treatment for all ECs is surgery, followed by adjuvant therapy if necessary (14). This treatment protocol is usually sufficient for the clinically indolent and hormone-responsive type I ECs, which comprise 80% of all ECs. However, the other 20% of ECs are aggressive type II cancers, with serous histology as the most prominent subtype (15, 16). Unfortunately, most of these high-grade tumors are resistant to conventional chemo- and radiation therapies, underscoring the major clinical need for improved treatment regimens for this EC subgroup (17–23). Additionally, due to their late stage detection and metastatic character, surgery is usually not an option for type II ECs, since the cancer has often already spread outside the uterus at the time of diagnosis (24–26). Therefore, not surprisingly, most EC-related deaths are due to the type II cancers, with dismal overall survival rates of generally <30% (25, 27–29).

Despite the above knowledge, patients with type II EC are still treated with largely ineffective chemotherapy regimens, thereby leading to unnecessary physical and economic burdens for the patient. In Japan, for example, one cycle of the commonly used carboplatin/paclitaxel protocol consists of three courses with a cost of ~2,000 Euros per course (30). Additionally, the medical care costs for managing the side effects were 1.6 times as much as the cost for one course, accumulating in a total cost of ~9,200 Euros per chemotherapy cycle. A study in the United Kingdom further demonstrated that the total costs (diagnosis/surgery, adjuvant therapy, and further treatment) increased with increasing EC grade (31). Hence, more pre-clinical studies and clinical trials should be focusing on targeted therapies in order to provide more adequate treatment options for patients with high-grade type II EC (32). This view seems particularly justified in light of the fact that successful results have already been obtained in other cancer types and several molecular markers have been identified in EC (33–35). One of the most promising markers in this context is certainly *PPP2R1A*, encoding the Aα subunit of the tumor suppressive phosphatase PP2A.

## The Tumor Suppressive Protein Phosphatase PP2A

Reversible phosphorylation is the key pillar on which signal transduction is built. The enzymes responsible for these post-translational modifications are the protein kinases, which catalyze phosphorylation of proteins, and the protein phosphatases, which, in turn, remove the phosphate group from their substrates (36–38). Importantly, the presence or absence of a phosphate can affect the biological activity of the modified protein, either positively or negatively, depending on the substrate. Like that, kinases and phosphatases act as on/off or off/on switches in cellular signaling. The balanced activities between both enzymes ensures that cellular homeostasis is preserved and that cells can generate the appropriate responses (e.g., proliferation, differentiation, survival, apoptosis…) to specific external stimuli. However, in cancer cells, this balance is genetically disrupted by mutations in key signaling molecules that often directly or indirectly affect kinases and phosphatases, so that signaling pathways will be constitutively activated or inhibited, eventually leading to overall uncontrolled cell growth and survival (39, 40).

Initially, in cancer research, protein kinases got the bulk of the attention, since their over-activation commonly drives oncogenic signaling and their pharmacologic inhibition showed promising clinical potential (6, 38, 41, 42). Furthermore, with more than 500 genes encoding protein kinases, they were thought to be the specific regulators of important oncogenic signaling pathways (43). However, if one accepts that a change in phosphorylation often just reflects an altered balance between kinase and phosphatase activities, kinases and phosphatases seem equally attractive therapeutic targets. Nevertheless, compared to kinases, phosphatase research, and phosphatase-directed therapies have lagged behind for a long time. This has in part been due to the fact that the first phosphatase was discovered 20 years after the first kinase, and that protein phosphatases were for a long time regarded as significantly less specific and less amenable to regulation by external stimuli, rendering them less attractive as therapeutic targets (44, 45). However, more and more attention has been brought to the phosphatases nowadays, hopefully resulting in more clinical applications in the near future (37, 46, 47).

**Figure 1:**
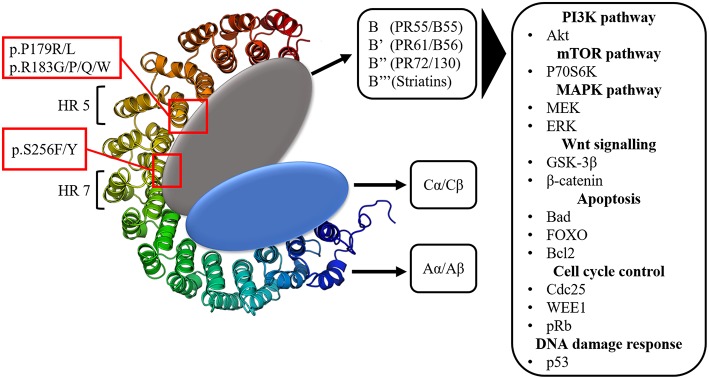
The structure of the protein phosphatase PP2A. Three subunits can be distinguished: the scaffolding A subunit (Aα/Aβ) composed of 15 heat repeats (HR), the catalytic C subunit (Cα/Cβ) and the 4 classes of regulatory B subunits. *PPP2R1A* hotspot mutations occur in HR 5 (p.P179R/L, p.R183G/P/Q/W) and HR 7 (p.S256F/Y), which are involved in B subunit binding. The many regulatory B subunits allow for PP2A to target a vast array of components of important signaling pathways often involved in tumorigenesis.

The large majority of protein phosphorylation occurs on serine (Ser) and threonine (Thr) residues (48). The Ser/Thr phosphatase PP2A constitutes about 1% of the total cellular protein content and is together with Protein Phosphatase 1 (PP1) responsible for more than 90% of all Ser/Thr phosphatase activity in the cell (49, 50). PP1 and PP2A are both holoenzymes, consisting of different subunits. PP2A consists of a dimeric core enzyme composed of a catalytic C subunit and a scaffolding A subunit (Figure 1). In humans, each of these subunits have two isoforms, α and β, of which the α isoform is the most commonly expressed in most cell types (51, 52). However, in order to target specific protein substrates, a third subunit needs to be associated with the AC core dimer, resulting in the formation of the trimeric PP2A holoenzyme (Figure 1). This third subunit is referred to as the regulatory B subunit and determines the subcellular localization and substrate specificity (53). The human genome encodes four different families of B subunits, which mutually exclusively bind the AC core dimer: PR55 (B/B55), PR61 (B'/B56), PR72/130 (B”), and B”' (Striatins). Furthermore, each family of B subunits consists of several isoforms (α up to ε) and splice variants, allowing for the formation of many different PP2A holoenzymes (53).

This huge structural diversity of PP2A holoenzymes forms the basis for its diverse functions in cellular signaling by allowing PP2A to act on various components within important signaling pathways (54). The main pathways affected by PP2A are the PI3K (Akt), mTOR (p70S6K) and MAPK (MEK/ERK) pathways (Figure 1). Additionally, PP2A also targets the oncoprotein cMyc as well as components involved in Wnt (GSK-3β, β-catenin) signaling, apoptosis (Bcl2, Bad, FOXO), cell cycle regulation (cdc25, WEE1, pRb) and DNA damage response (p53, ATM, Chk) (51, 55–60). All of these pathways are key regulators of processes imbalanced in tumorigenesis (e.g., protein synthesis, cell proliferation, cell survival, cell migration, and invasion). Since PP2A usually negatively affects these pathways, it was denoted as a potential tumor suppressor (61).

The tumor suppressive properties of PP2A were first demonstrated in *in vitro* experiments using the tumor promoting agent and selective PP2A inhibitor, okadaic acid (OA), as well as the simian virus 40 (SV40) small T antigen. These experiments showed that PP2A inactivation is an absolute requirement in order to achieve oncogene-induced transformation of immortalized human epithelial cells (e.g., by oncogenic H-*Ras*). OA is able to inhibit PP2A by acting on the catalytic C subunit, while SV40 small T antigen inhibits PP2A by binding the Aα subunit, thereby replacing specific B subunits (61–64). The tumor suppressive nature of PP2A was further corroborated by *in vivo* evidence in mice. For example, mice completely lacking the B56δ subunit, or showing ≥50% decreased expression of the phosphatase 2A phosphatase activator (PTPA), spontaneously developed tumors (65–67). Additionally, the general physiologic importance of PP2A function was demonstrated in several mouse models (68). For example, PP2A Aα or PP2A Cα knock-out mice are embryonically lethal, indicating the importance of PP2A already during development. Furthermore, a vast array of pathological phenotypes were observed in mice with different genetic PP2A dysfunctions, stressing the importance of functional PP2A in many crucial signaling pathways and in tissue homeostasis (68).

In line with these studies, many human cancers have shown to be associated with PP2A dysfunction (69–71). The main mechanism of PP2A inactivation in cancer is via the overexpression of the endogenous PP2A inhibitors SET (Suvar/Enhancer of zeste/Trithorax) and CIP2A (Cancerous Inhibitor of PP2A) (69, 72, 73). However, PP2A can also be inactivated via aberrant post-translational modifications, mostly via PP2A methylesterase (PME-1) upregulation, thereby stabilizing inactive PP2A complexes through binding and/or demethylation of the C subunit C-terminal tail (74–76). Another way of PP2A inactivation is via mutations in one of its subunits (77), or via mutations or heterozygous loss of the cellular PP2A activator PTPA (*PPP2R4*) (66). Interestingly, PP2A dysfunction is very common in endometrial cancer, as well as in other gynecologic malignancies, such as ovarian and cervical cancer. In the following part, we will give an overview of how PP2A is specifically inactivated in these gynecologic cancers.

## The PP2A Status in Endometrial and Other Common Gynecologic Cancers

### PP2A Status in Endometrial Cancer

Inactivation of PP2A is observed in both type I and type II ECs. However, the way PP2A is inactivated seems to be quite different in both EC subtypes.

In type I endometrioid ECs, PP2A inactivation is likely indirect via an upregulation of the endogenous PP2A inhibitors CIP2A or PME-1. Immunohistochemical analysis of paraffin-embedded EC tissue revealed positive CIP2A staining in 79% of the cases. Additionally, increased CIP2A mRNA levels were observed in fresh human EC tissue compared to healthy endometrial tissue (78). CIP2A depletion in endometrioid cancer cells decreased cell proliferation and invasion, while apoptosis was increased, indicating the oncogenic role of CIP2A in type I EC and its potential as a therapeutic target (78). Detailed information on the mechanism of CIP2A overexpression in ECs is still lacking. However, in estrogen receptor (ER)-positive breast cancer cells, estradiol (E2) was able to increase CIP2A protein levels through the ERα (79). Therefore, it can be hypothesized that the same could be true for the estrogen-dependent type I ECs. Additionally, it could be one of the explanations why CIP2A overexpression is rare in the estrogen-independent type II ECs.

In addition, Wandzioch et al. demonstrated PME-1 overexpression in endometroid EC cell lines as well as patient samples (80). PME-1 expression was about 20 times higher in tumor tissue compared to healthy tissue, indicating PME-1 could also be a new potential biomarker for type I ECs. In case of PME-1 upregulation, PP2A activity was significantly reduced, resulting in an increased oncogenic phenotype via upregulation of the PP2A targets Akt and ERK (80).

In contrast to type I ECs, PP2A inactivation in type II ECs is, to our knowledge, not associated with CIP2A or PME-1 overexpression. Instead, up to 40% of type II EC tumors are associated with heterozygous missense mutations in *PPP2R1A* (34, 35, 81–95). *PPP2R1A* mutations also occur in type I ECs, albeit at very low frequencies (2.5–6.9%) (32).

**Figure 2:**
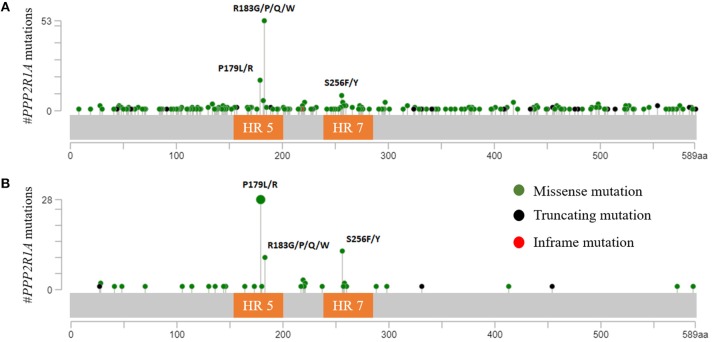
**(A)** *PPP2R1A* mutations across all available cBioportal cancer studies except ECs. The three hotspot mutations (p.P179; p.R183; and p.S256) are clearly depicted. Most tumors (66.3%) had mutations in p.R183 while only 22.5% and 11.3% had mutations in p.P179 and p.S256, respectively. **(B)** *PPP2R1A* missense mutations reported in type II ECs (cBioportal). In contrast with other cancers, *PPP2R1A* mutations mostly occur at residue p.P179 (56%), while only 20% had mutations in residue p.R183. Also more EC tumors (24%) had mutations in p.S256 compared to other cancer types. HR, HEAT-repeat.

*PPP2R1A* encodes the Aα subunit of PP2A and is an established tumor suppressor gene (96, 97). The structure of Aα is characterized by 15 Huntingtin-Elongation-A subunit-TOR (HEAT) repeats (98) (Figure 1). Each of these HEAT repeats consists of a pair of anti-parallel alpha helices connected via intra-repeat loops. These intra-repeat loops are responsible for the interaction with the C and B subunits. More precisely, HEAT repeats 1–10 are able to bind the regulatory B subunits while HEAT repeats 11–15 bind the catalytic C subunit. Remarkably, most of the *PPP2R1A* mutations cluster together in HEAT repeats 5 and 7, which are involved in B subunit binding. Another intriguing fact is that these *PPP2R1A* mutations almost always occur at the same residues across several cancer types, forming so called hotspot mutations. These hotspot mutations include p.P179R/L, p.R183G/P/Q/W (HEAT repeat 5), and p.S256F/Y (HEAT repeat 7) (93, 94, 99–101). Remarkably, in type II EC, p.P179L/R hotspot mutations are a lot more abundant than in most other cancer types (Figure 2). Biochemical studies in endometrial cancer cells demonstrated that C subunit binding for these hotspot mutations was significantly reduced. Strikingly, loss of C subunit binding was more severe for p.P179R (80% less C binding compared to wild type) than for p.R183W and p.S256F (50–60% less C binding compared to wild type). This biochemical difference between the hotspot mutations could result in distinct functional consequences, although further research is needed to investigate this. Moreover, *PPP2R1A* hotspot mutations resulted in deficient binding to specific regulatory B subunits (B55α, B56α,β,ε, B”/PR72), while some B subunits (i.e., B56δ and B56γ, and the striatins) retained binding to the mutated Aα subunit (102, 103). Nevertheless, despite the retained binding of these B subunits, the phosphatase activity of the Aα mutated PP2A-B56δ, γ trimers was reduced. Therefore, it was suggested that *PPP2R1A* mutations are not simply loss-of-function (i.e., by their inability to bind specific B-type subunits), but may also lead to a dominant-negative inhibition of specific PP2A complexes (i.e., by their ability to retain binding to specific B-type subunits, but capturing them in trimeric complexes with decreased phosphatase activity). This hypothesis was further mechanistically underbuilt by mass spectrometry-based identification of the (mutant) Aα interactomes. These interactomes indeed revealed that the Aα mutants had an increased binding to the endogenous PP2A inhibitor TIPRL1, which could explain the decreased PP2A activities of retained mutant Aα-PP2A trimeric complexes (102). In accordance, ectopic expression of several Aα mutants, among which p.P179R and p.S256F, in the wild-type *PPP2R1A*-expressing HEC-1A EC cell line, resulted in increased anchorage-independent cell growth and increased xenografted tumor growth in nude mice, and correlated with increased Akt and mTOR/S6K oncogenic signaling (102).

Besides the *PPP2R1A* mutations, also mutations in *PPP2R1B*, encoding the PP2A Aβ isoform, have been reported for ECs in the cBioportal database, albeit with occurrences of <1% (mainly deletions) in both EC subtypes (93, 94).

### PP2A Status in Ovarian and Cervical Cancer

Inactivation of PP2A has also been reported in other gynecologic cancers. In contrast to type II ECs, and similar to type I ECs, ovarian (0–7%), and cervical cancers (0–1%) hardly present any mutations in *PPP2R1A* (91, 93, 94, 104–112). Interestingly, however, the few number of *PPP2R1A* mutations that are present mainly occur at the hotspot residue p.R183, which is most frequently affected across all cancer types (Figure 2A).

Likewise, mutations in *PPP2R1B*, were also reported in ovarian and cervical cancers, albeit at very low frequencies, and without any hotspot mutations. Furthermore, the presence of *PPP2R1B* mutations or loss of heterozygosity was not relevant for ovarian and cervical tumorigenesis (113–116).

Instead, the main way of PP2A inactivation in both ovarian and cervical cancers is indirect. Indeed, a retrospective analysis of serous ovarian cancer demonstrated 40.4% of the specimens to have strong CIP2A immunoreactivity and another 42.4% had weak positive staining (117). Another retrospective study of 152 ovarian cancer specimens (serous, endometrioid, mucinous and clear cell) further corroborated this by showing CIP2A overexpression in 65.79% of samples tested (118). Likewise, recent studies have shown SET overexpression in ovarian cancers (119).

Increased CIP2A expression levels have also been reported in cervical cancer. For example, one study reported on the expression of CIP2A in 60.8% of samples from patients with squamous cervical cancer while this was only 5.7% in normal cervical epithelial tissue. Furthermore, five cervical cancer cell lines harbored elevated CIP2A levels (120). This was further corroborated by a study of Huang et al. in which CIP2A expression was observed in cervical cancer cell lines but not in normal epithelial cells. Additionally, cervical cancer tissue had higher CIP2A mRNA levels compared to healthy adjacent tissue (121). In cervical cancer, CIP2A is mainly upregulated via the E6 and E7 oncoproteins expressed by the human papilloma virus (HPV) type 16 (122–124), the most common type of HPV in cervical cancers (125). In addition to the indirect PP2A inhibition via CIP2A, Pim et al. also proposed a direct way of PP2A inhibition in cervical cancers. They observed that the E7 oncoprotein is able to bind the PP2A Aα and Cα subunits, thereby displacing the B subunit. This way E7 is probably acting in the same way as the SV40 small T antigen rendering PP2A unable to dephosphorylate and inhibit its oncogenic targets (126, 127). In contrast, however, White et al. were not able to demonstrate this interaction between the E7 oncoprotein and PP2A, despite some similarities between E7 and the SV40 small T antigen (128). Finally, another way of decreased PP2A activity during cervical carcinogenesis might be via reduced PP2A Cα expression, potentially by a microRNA-dependent mechanism (129).

Summarized, based on the mechanism of PP2A inactivation, two groups can be distinguished within gynecologic cancers. The first group comprises type I endometrioid EC, ovarian and cervical cancers and is characterized by PP2A inactivation mainly via CIP2A or PME-1, and perhaps also via SET. This group also has a very low frequency of *PPP2R1A* mutations. However, when present, these mutations recur mostly at hotspot residue p.R183. The second group comprises the type II ECs. This cancer type lacks CIP2A and PME-1 overexpression, but harbors frequent heterozygous missense mutations in *PPP2R1A*. Moreover, these missense mutations are different from the ones associated with the first group. More precisely, *PPP2R1A* mutations in type II ECs most frequently recurred at residues p.P179 and p.S256 (Figure 2B). Indeed, when looking at the cBioportal database, almost all hotspot mutations in p.P179 and p.S256 were associated with type II ECs, while hotspot mutations in p.R183 are more frequently observed across other cancer types (Figure 2A) (90, 93, 94, 130). The reasons for this distinct mutational pattern remain currently unclear. However, distinct *PPP2R1A* missense mutations might affect B subunit binding and PP2A activity in a slightly different way (102), thereby, in part, contributing to a different tumor biology in type I and II ECs as well as other cancers. However, further research is warranted in order to fully understand the involvement of specific PP2A holoenzymes in different cancers. Nevertheless, the distinct PP2A inactivating mechanisms between type I and type II ECs, as well as other gynecologic cancers, open up specific opportunities for direct or indirect, personalized therapeutic targeting of PP2A, in order to (re)-activate this phosphatase.

## PP2A as a Potential Therapeutic Target in Endometrial Cancer

In the last few years, the notion that phosphatases as opposed to kinases could also be useful therapeutic targets has gained more and more attention (54, 131–134). In contrast to therapeutic inhibition of kinases, the focus with targeting phosphatases, and in particular the tumor suppressive phosphatase PP2A, is on the development of activating or reactivating compounds (133). These compounds are either able to relieve the inhibition by endogenous inhibitors, or directly bind and activate PP2A. In contrast, some studies also reported on the benefit of PP2A inhibition rather than activation. Despite the promising therapeutic potential of these compounds in multiple pre-clinical studies in several other human cancer types, pre-clinical studies, specifically in EC, are however, mostly lacking. In the following four subsections, we will provide an overview of the most promising PP2A-targeted therapies, which—pending additional dedicated studies—may become applicable to EC as well.

### Compounds Indirectly Activating PP2A

#### CIP2A Targeting Compounds

Since CIP2A is commonly overexpressed in several cancers, it has become an interesting therapeutic target in order to re-activate PP2A. Specifically in type I EC, CIP2A depletion decreased proliferation and invasion, and increased apoptosis *in vitro* (78), indicating it could be a valuable therapeutic target. Furthermore, depletion of CIP2A also showed anti-tumorigenic potential in ovarian and cervical cancer cells (118, 120, 135).

Most of the currently described CIP2A targeting compounds are able to increase PP2A activity by reducing the CIP2A protein levels, either via downregulation of CIP2A expression or by promoting its degradation. In cervical and endometrial cancer cells, CIP2A expression is mainly regulated by two transcription factors, Elk1 and Ets1, which are both necessary for regulating CIP2A protein levels (136). Additionally, in cervical cancer, the transcription factor E2F1 has also been implicated in the regulation of CIP2A expression via the E7 oncoprotein (124, 137). Therefore, compounds targeting one of these factors could have potential therapeutic value. For example, several erlotinib derivatives were able to reduce CIP2A levels and increase PP2A activity in breast cancer and hepatocellular carcinoma cells via disrupting the interaction between the transcription factor Elk1 and the *CIP2A* promotor (138, 139). On the other hand, lapatinib downregulated CIP2A through regulation of protein stability in breast cancer cells (140). Also, bortezomib, a US Food and Drug Administration (FDA)-approved proteasome inhibitor, was able to reduce CIP2A expression levels in several cancer cell lines, although the mechanism of action is not elucidated yet (141–143). Furthermore, increased CIP2A degradation through autophagy was seen in breast cancer cells upon mTORC1 inhibition (e.g., using rapamycin) (144). Additionally, several natural compounds have demonstrated PP2A re-activating potential via downregulation of CIP2A. Despite the lack of studies testing these compounds in EC, positive results have already been obtained in other cancer studies. For example, rhabdocoetsin B, arctigenin and the red wine component ellagic acid were able to reduce CIP2A transcription levels in breast and lung cancer cells (145–147). On the other hand, celastrol and gambogenic acid promoted CIP2A degradation in lung and liver cancer cells (148, 149). Additionally, the compounds genistein and ethoxysanguinarine promoted both transcriptional suppression and proteasomal degradation of CIP2A (150, 151). Lastly, fusogenic-oligoarginine peptide-mediated delivery of siRNA targeting CIP2A has also appeared as a new therapeutic strategy, showing anti-tumorigenic potential *in vitro* and *in vivo* in oral cancer cells (152, 153). It would be extremely interesting to test whether any of these known CIP2A inhibiting compounds would have therapeutic benefits in CIP2A-overexpressing endometrioid EC models.

#### PME-1 Targeting Compounds

PME-1 has also emerged as a potential therapeutic target in endometrioid EC, especially since PME-1 depletion using RNA interference resulted in increased PP2A activity, thereby reducing the oncogenic phenotype of type I EC cells *in vitro* and in xenograft assays (80). Additionally, PME-1 depletion in HeLa cells, a cervical cancer cell line, also led to decreased proliferation and colony formation by increasing PP2A activity and thereby inhibiting MAPK pathway activity (154).

So far, two classes of pharmacologic PME-1 inhibitors have been discovered, the ABL (Aza-β-lactam) inhibitors and the sulfonyl acrylonitrile inhibitors, which both irreversibly bind to PME-1 and inhibit PME-1 esterase activity (155, 156). Pusey et al. tested two of these PME-1 inhibitors, ABL-127 and AMZ-30, of which ABL-127 was the most potent one in EC models. However, *in vivo* testing of this compound in xenograft assays could not corroborate the *in vitro* data, implying that inhibition of solely the PME-1 esterase activity may be insufficient to inhibit PME-1's oncogenic characteristics (157).

#### SET Targeting Compounds

Although the relevance of SET overexpression (if any) in EC is currently unclear, SET inhibitors can mainly be divided into three groups according to their origin. The first group comprises sphingolipid-based compounds, such as ceramide and FTY720 (also called Fingolimod), as well as their derivatives. The second group resembles the apolipoprotein E (ApoE) and these compounds are denoted as SET interfering peptides (e.g., COG112 and OP449). More recently, potent cytotoxic effects were reported for cell penetrating peptides, the third group of SET inhibitors, which constitute the precise SET-PP2A interaction interface (158). Although the mechanism of action of all these SET inhibitors is not always well understood, they most likely increase PP2A activity in the same way, i.e., via disruption of the interaction between SET and PP2A (53, 59, 133). Recently, the interaction between the sphingolipid-based compounds (i.e., ceramide and FTY720) and PP2A were investigated in more detail using NMR spectroscopy (159). In this study, they observed that the sphingolipid compounds probably work by disrupting the dimerization of SET, which is thought to be important for its PP2A binding and inhibiting activity.

### Compounds Directly Activating PP2A

Recently, small molecule activators of PP2A (SMAPs) have been developed (160). These SMAPs are derived from the anti-psychotic phenothiazines and are predicted to directly activate PP2A. Although a direct binding to the PP2A Aα subunit has been demonstrated (161), the mechanism by which these SMAPs are able to activate PP2A remains unknown. Nevertheless, several pre-clinical studies have shown the promising anti-proliferative potential of SMAP treatment, for example in T-cell acute lymphoblastic leukemia, castration-resistant prostate cancer, KRAS mutant lung adenocarcinoma, and tyrosine kinase inhibitor (TKI)-resistant lung adenocarcinoma (161–164). Hence, it would be of amazing interest to test these promising compounds in pre-clinical EC models with intact as well as impaired PP2A functionality.

### The Therapeutic Potential of Combination Therapies

Besides the therapeutic potential of single agent targeting of PP2A, also combination therapies of PP2A activators with other drugs have gained attention (47, 165). The combination of a PP2A activator with a kinase inhibitor seemed particularly beneficial in cases where oncogenic kinase activation simultaneously resulted in PP2A inhibition, and therapy resistance to a single agent kinase inhibitor occurred (166–168). For example, *KRAS-*mutant lung cancer and pancreatic ductal adenocarcinoma cells showed resistance to MEK inhibitors and mTOR inhibitors, respectively. This resistance occurred due to cross-talk with the PI3K/Akt/mTOR pathway and cMyc oncoprotein upregulation, probably via PP2A inhibition. Hence, they tested the combination of a direct PP2A activator (SMAP) with a MEK or mTOR inhibitor, which resulted in significantly increased anti-cancer effects *in vitro* as well as *in vivo* (169, 170). Such combinatorial benefit was further demonstrated in myeloid leukemia where the combination of a SET inhibitor (indirect PP2A activation) with a tyrosine kinase inhibitor resulted in synergistic anti-cancer effects (171, 172). In TKI-resistant lung adenocarcinoma cells, the synergistic effects of a SMAP and the TKI afatinib were in part also contributed to a downregulation of CIP2A (164).

The combination of PP2A activators with chemotherapy has also been investigated, although not yet in EC. Several studies tested the effect of combining SET inhibitors (FTY720, OP449) with different chemotherapy regimens (e.g., doxorubicin, cisplatin). These studies demonstrated synergistic anti-cancer effects in myeloid leukemia, breast cancer cells, colorectal cancer cells as well as in cisplatin-resistant melanoma and lung cancer cells (171, 173–175).

Overall, these successful precedents open up possibilities to test these PP2A activating compounds in EC models, possibly in combination with kinase inhibitors or chemotherapeutics. This could be specifically interesting for the type II serous ECs, in which therapeutic combinations with PP2A activators might sensitize these cancer cells toward the current, mainly ineffective, therapies.

### Exploiting PP2A Inhibition for Therapeutic Purposes

In contrast to the therapeutic potential of PP2A activation, some studies also reported on the therapeutic relevance of PP2A inhibition, when applied together with a DNA damaging treatment, or when combined with immunotherapy (176, 177). The anti-cancer effect of PP2A inhibition in combination with DNA damaging agents can be explained by the enabling role of PP2A in DNA damage response and repair pathways as well as in cell cycle regulation. Hence, PP2A inactivation in this situation (i.e., combined with chemo-or radiation therapy) leads to aberrant cell cycle progression and checkpoint activation, resulting in mitotic catastrophe and, consequently, cell death (133, 178, 179). Likewise, PP2A is also involved in the immune response by negatively regulating the function of cytotoxic T-lymphocytes (176, 180). Therefore, PP2A inhibition combined with immunotherapy could enhance the immune-mediated anti-tumor response.

The small molecule LB-100 is one of the best studied PP2A inhibitors so far, without any apparent toxicities in animals and with promising results in a first human clinical trial (181, 182). Pre-clinical studies demonstrated LB-100 was able to sensitize many different solid tumor cells to DNA damaging agents. For example, LB-100 enhanced cisplatin-mediated cytotoxicity in ovarian carcinoma cells *in vitro* and *in vivo* in xenografts (178, 183, 184). Likewise, the combination of LB-100 with the immune checkpoint inhibitor aPD-1 in colon and melanoma cancer cells resulted in an enhanced and durable T-cell-dependent anti-tumor response, with more effector T-cell and less suppressive regulatory T-cell infiltration (176). On a critical note, it needs to be mentioned here though, that recent evidence has suggested that LB-100 is not entirely specific for PP2A, and also inhibits the catalytic activity of the related Ser/Thr phosphatase PP5 (182). As PP5 is considered as tumor promoting, PP5 inhibition could contribute to the anti-tumor activities of LB-100. This was further corroborated *in vitro* in ovarian cancer cells, where knockdown of PP5 resulted in decreased cell proliferation and colony formation.

To conclude, further research is warranted to fully understand how both PP2A activation and inhibition can be therapeutically viable as anti-cancer treatment for EC.

## PP2A Dysfunctions as Predictive Biomarkers for Targeted Therapies in EC

### CIP2A-Mediated PP2A Inhibition

So far, a wealth of studies reported on the role of CIP2A overexpression, and thereby likely PP2A inactivation, as a potential predictive biomarker for diverse therapies (targeted and untargeted), in a large variety of solid cancers (54). For example, in lung and breast cancer, the overexpression of CIP2A resulted in resistance to the EGFR inhibitors lapatinib and erlotinib, while RNA interference-mediated CIP2A depletion sensitized the cells toward these compounds (140, 185). CIP2A overexpression also resulted in resistance toward Chk1 kinase inhibitors in gastric adenocarcinoma and breast cancer cells (186). Furthermore, CIP2A overexpression conferred resistance to chemotherapy in several solid tumor types, including cervical and ovarian cancers (187–190). Liu et al. further demonstrated that in HeLa cells, treated with several chemotherapeutics such as paclitaxel, doxorubicin and cisplatin, CIP2A expression was significantly associated with drug insensitivity through increased expression of p-glycoprotein drug efflux pumps (190). Additionally, the use of siRNA, targeting CIP2A *in vitro*, resulted in sensitization of HeLa cells to different chemotherapeutics (190). Finally, the natural CIP2A inhibitors, ethoxysanguinarine and gambogenic acid, sensitized lung and hepatocellular cancer cells to chemotherapy (149, 151).

While there is a general lack of studies on the predictive potential of CIP2A expression in EC models, it is very likely that, based upon the evidence obtained in other solid (gynecologic) tumors, CIP2A expression could mediate therapeutic resistance in EC cells as well. This further implies that CIP2A status of the EC tumors should better be taken into account in clinical trial set-ups. Additionally, the data illustrate the potential advantages of combining PP2A activators with EC therapies that are mainly ineffective on their own.

### Recurrent *PPP2R1A* Hotspot Mutations

Whether *PPP2R1A* mutations are present in EC tumors or not, could have consequences for the efficacy of targeted therapies. For example, kinase inhibitors targeting the PI3K/Akt/mTOR pathway or MEK/MAPK pathway, commonly affected in ECs, could be less effective when PP2A, counteracting the targeted kinase, is mutated. The rationale behind this is that a kinase inhibitor can only work to its full potential when the opposing phosphatase is not inactivated. In case the phosphatase is dysfunctional, the net phosphorylation would be largely unaffected and the pathway would remain activated. Simply put, the use of certain kinase inhibitors in case of PP2A dysfunction would be the equivalent of pouring water into a bucket with holes in it. This biochemical logic was nicely underscored by Kauko et al. who showed that PP2A inhibition achieved by siRNA-mediated knockdown of Aα, conferred resistance to a MEK inhibitor in *KRAS-*mutant lung cancer cells (169).

On the other hand, *PPP2R1A* mutations in type II ECs could also be predictors of positive outcome to certain kinase inhibitors. For example, Haesen et al. showed hyperactivation of the PI3K/Akt/mTOR pathway in *PPP2R1A* mutated EC cells, while the MAPK pathway was actually downregulated (102). This indicates single agent kinase inhibitors targeting the PI3K pathway might be effective in *PPP2R1A* mutated ECs, since cross-talk to the MAPK pathway would possibly be absent and *PPP2R1A* mutant EC cells might be dependent on PI3K/Akt/mTOR signaling for growth and survival (32). Patient stratification based on *PPP2R1A* status of the tumor could also be applied to other gynecologic cancer types, even when *PPP2R1A* mutations are rare. For example, Papp et al. demonstrated ovarian cancer cell lines harboring *PPP2R1A* mutations to be more sensitive to a PI3K/mTOR inhibitor (108). However, in this experiment they took cell lines with mutations in *PPP2R1A* and *PARP1* into account, which could bias the results.

Finally, the response of type II ECs to SMAPs could also be dependent on the *PPP2R1A* mutational status of the cancer cells, especially since these SMAPs bind to the Aα subunit in close proximity to the *PPP2R1A* hotspot mutations (161). Therefore, mutations in this subunit could disturb the interaction with the SMAPs and consequently render the compound ineffective. Targeted pre-clinical studies addressing these possibilities should provide further insights in these issues in the near future.

### Others

Although no studies have yet addressed the predictive role of PME-1 overexpression in type I EC, overexpression of PME-1 in glioma drives resistance to various multikinase inhibitors. Consequently, PME-1 depletion resulted in enhanced sensitivity to these inhibitors *in vitro* and *in vivo* in xenografts (191).

As identified through a large siRNA screen, decreased expression of the PP2A activator PTPA conferred significantly increased resistance of cervical HeLa cells to several cytotoxic agents, including cisplatin, taxol, and etoposide (192), perhaps suggestive for a similar dismal predictive role for heterozygous loss or mutation of PTPA in type II endometrial carcinosarcoma (66).

Likewise, increased SET expression has been associated with resistance to TKI's, cisplatin, paclitaxel, oxaliplatin, and 5-fluoro-uracil in diverse cancer types (54, 193, 194). Whether this would be relevant for EC, remains, again, to be determined.

## PP2A Dysfunctions as Prognostic Biomarkers for EC

### CIP2A Overexpression

In several cancers, PP2A inactivation is associated with significantly worse prognosis (54). For example, overexpression of the PP2A inhibitor CIP2A is correlated with worse prognosis in several solid tumors as well as in myeloma (195–197). Specifically, for gynecologic cancers, several studies reported on the prognostic potential of CIP2A in cervical and ovarian cancer. In serous ovarian cancers, strong CIP2A immunoreactivity correlated with worse prognosis (117), and the same was observed in a retrospective study on 152 ovarian cancer specimens, including serous, endometrioid, mucinous, and clear cell subtypes (118). In cervical cancer, CIP2A was found to associate with H-Ras to promote epithelial-mesenchymal transition, resulting in increased migration and invasion of cervical cancer cells *in vitro* and *in vivo* (120). Furthermore, cervical cancer tissue analysis revealed that CIP2A expression correlated with lymph node metastasis and high-grade and advanced stage cervical cancer (120). However, these results are in contrast with the human protein atlas database (www.proteinatlas.org) which does not put CIP2A forward as an unfavorable prognostic marker in both ovarian and cervical cancers (198).

Data concerning the prognostic potential of CIP2A in EC are scarce. One study of Yu et al. demonstrated that CIP2A expression in type I endometrioid EC correlated with increased FIGO stage and tumor grade (78). Furthermore, according to the human protein atlas, CIP2A expression correlated with worse prognosis in patients with EC. Nevertheless, more studies are necessary to further demonstrate the prognostic potential of CIP2A in EC.

### Recurrent *PPP2R1A* Hotspot Mutations

**Figure 3:**
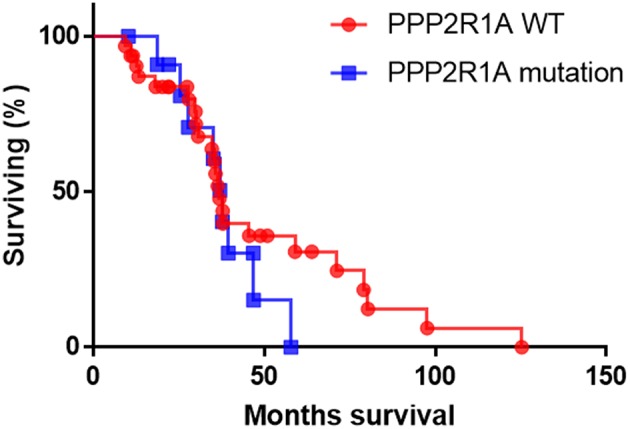
The overall survival of patients with type II serous EC with (blue line) and without *PPP2R1A* mutations (red line). (*n* = 44), data were extracted from the UCEC-TCGA study available in the cBioportal database.

In the absence of any published work on the prognostic marker potential of *PPP2R1A* in type II EC so far, we used the cBioportal database to analyze the survival data of type II ECs. However, survival data were only available for 44 patients (UCEC-TCGA study) with type II serous EC, of which only 12 presented with mutations in *PPP2R1A* (93, 94). Analysis of this limited data set revealed no significant difference in overall survival between patients with and without *PPP2R1A* mutations (*P* = 0.39) (Figure 3). However, more patient data are definitely required in order to obtain more conclusive results. Longer patient follow-up and centralized data collection in multi-institutional centers could boost the data collection for patients with this rare endometrial cancer subtype.

### Others

Similarly, PP2A inactivation via *PPP2R4* (PTPA) haploinsufficiency leads to a worse prognosis in many cancer types, including endometrial carcinosarcomas (66). Also, lower PP2A/C expression in cervical cancer was closely associated with the nodal status of cervical cancer patients (129).

Although there is a lack of studies on the role of SET in EC, the protein atlas database (www.proteinatlas.org) indicates that there is no correlation between SET expression and a worse prognosis in gynecologic cancers (198). On the other hand, a strong correlation was seen between SET expression levels and decreased survival of ovarian cancer patients (119).

## Future Steps

In this review, we discussed the therapeutic potential of PP2A targeting as well as the biomarker potential of PP2A dysfunctions in EC, and other gynecologic cancers. Specifically, in type II EC, *PPP2R1A* mutations are remarkably common, while in type I EC, PP2A dysfunction rather occurs through non-genomic mechanisms, involving increased expression of PP2A inhibitors CIP2A and PME-1. However, in order for *PPP2R1A* to become a clinically relevant biomarker for type II ECs, reliable and fast detection of somatic mutations in this gene will be necessary. Therefore, future development of methods able to detect somatic mutations in tumor samples, or preferably in liquid biopsies, will be crucial.

Over the last few years, several promising methods have been developed for the detection of mutations in oncogenes. For example, Spaans et al. designed a Matrix-assisted laser desorption/ionization time-of-flight mass spectrometry (MALDI-TOF) based somatic mutation panel able to detect hotspot mutations in 13 genes frequently mutated in gynecologic cancers (199). This method is able to provide reproducible, high-throughput data based on low quality and quantity DNA from formalin-fixed paraffin-embedded (FFPE) samples. Additionally, next-generation sequencing (NGS) has come forward as a potential technique to detect mutations in the clinic. NGS is able to rapidly detect all mutations in the complete gene of interest. However, data analysis is still rather complex and differentiating between somatic passenger and driver mutations can be time consuming. However, optimizations of this technique could lead to a more user-friendly method for the detection of specific mutations. For example, Cottrell et al. validated a NGS assay (WuCaMP) which targets a specific panel of genes with known clinical importance (200). This highly specific and sensitive assay allowed for a fast analysis of the target genes, thereby reducing time and costs of NGS.

More recently, also non-or minimally-invasive liquid biopsies have been investigated as a way to detect somatic mutations in patients with EC (201). For example, circulating tumor cells (CTCs) and circulating tumor DNA (ctDNA) extracted from blood samples or uterine aspirates showed potential as a way to screen for somatic mutations. However, whether CTCs can be useful for patients with high-risk EC is still unclear due to its debatable prevalence in blood samples. Bogani et al. reported a low prevalence of CTCs in pre-operative blood samples of patients with high-risk EC and even an absence of CTCs in patients with type II EC (202). In contrast, Alonso-Alconada et al. demonstrated the presence of CTCs in patients with high-risk EC (203). Thus, further research is warranted in order to prove the potential usefulness of CTCs in type II ECs. On the other hand, also cell-free DNA can be used to detect mutations. NGS was able to detect specific endometrioid EC-associated mutations in the cell-free DNA derived from peripheral blood samples of patients with early and late stage endometrioid EC (204). These promising results indicate the potential of this technique for the detection of type II ECs, when implementing *PPP2R1A* in the targeted gene panel.

Furthermore, also DNA obtained during a routine Pap (Papanicolaou) test can be analyzed for the detection of oncogenic mutations. For example, Wang et al. designed a test called PapSEEK, which is able to detect mutations in 18 commonly mutated genes in endometrial cancer (205). This is particularly interesting since with this method, oncogenic mutations could be detected already during a routine check-up, making early stage detection of (type II) EC possible. This is especially important since patients with type II EC show few symptoms until the disease is already in late stages and therefore less amenable to the current therapies.

In contrast, detection of CIP2A or PME-1 overexpression in (type I) EC cannot rely on genetic methods, but should rather focus on the mRNA or protein level, and is therefore, much more dependent on the availability of tumor biopsies or resected tumor material. In this respect, the use of reliable, validated antibodies, able to specifically detect these oncoproteins via immunohistochemistry techniques is crucial. However, the fact that CIP2A is always found expressed at high levels in the tumor tissue, while being nearly undetectable in the corresponding non-proliferating normal tissue, virtually eliminates the issues associated with the poorer suitability of immunohistochemistry as a technique to reliably quantify protein expression in tissues. In addition, the use of autoantibodies as serum biomarkers for CIP2A showed promising results in breast cancer patients (206). Therefore, it might also be interesting to investigate this in the setting of EC.

Despite the identification of the phosphatase PP2A as a promising molecular marker for ECs, few pre-clinical studies have investigated its potential as a direct therapeutic target, nor as a stratification marker for targeted kinase inhibitor treatments in this cancer type. Nevertheless, a plethora of studies in other solid tumor types suggest PP2A to have potential as a new therapeutic target for both type I and, more importantly, for the more aggressive type II ECs. We put several potential therapeutic compounds forward that could be tested in EC studies, potentially in combination with chemotherapy or targeted therapy.

So far, hormonal intervention and the immunotherapeutic pembrolizumab are the only two FDA-approved targeted therapies for hormone-dependent type I ECs, while there are none for the type II ECs (207). Nevertheless, molecular analyses of ECs have revealed that, in the large majority of ECs, the PI3K pathway is overactivated, which led to a number of (pre-)clinical studies investigating kinase inhibitors targeting this pathway (e.g., several mTOR and PI3K inhibitors) (32). The outcome of these studies was largely disappointing, not only due to the development of inherent resistance mechanisms (e.g., cross-talk to MEK/MAPK pathway), but also in a big part due to the complete lack of patient stratification in clinical studies (208, 209). The importance of the latter was further illustrated by the clear therapeutic benefit of the HER2 inhibitors trastuzumab (anti-HER2 antibody) and lapatinib (tyrosine kinase inhibitor, TKI), in type II serous EC cells stratified based on *HER2* amplification vs. normal *HER2*, or based on *HER2* amplification and functional PI3K vs. those with *HER2* amplification and mutant *PIK3CA* (32, 210, 211). Likewise, we hypothesized here that the PP2A status of the endometrial tumor, should be an important additional stratification marker for testing these targeted kinase inhibitors, given that PP2A mainly acts as a negative regulator of PI3K and HER2 downstream signaling, and hence its functional or dysfunctional state could co-determine kinase inhibitor therapy outcome. In case dysfunctional PP2A would mediate therapy resistance, the use of SMAP combination therapy could be a valuable solution. Also type I ECs could benefit from the combination of PP2A activators with standard therapies, since sensitization of the cells to chemo- or radiation therapy could result in a lower dose and duration of the therapy, required to treat type I ECs. This in turn, would reduce the physical and economic burden associated with chemotherapy.

Furthermore, since the mechanism of PP2A inactivation is different between both subtypes of EC, it will be important to stratify patients in those having type I and those having type II tumors within (pre)-clinical trials. This way, existing, or new therapeutic compounds will be tested on a more rational basis and no bias will occur toward the biggest group of indolent type I ECs. Therefore, the presence of certain PP2A dysfunctions in the EC tumor could indicate whether the patient is eligible for certain (targeted) therapies.

In conclusion, we highlighted the therapeutic potential of PP2A activating as well as inactivating compounds in several gynecologic cancers. However, it has to be noted that more studies should focus on these promising compounds in the specific context of type I and type II ECs. Furthermore, we demonstrated, based on studies in several other cancers, among which ovarian and cervical cancer, that PP2A dysfunction, due to mutations or cellular PP2A inhibitors, could be an indicator for worse prognosis as well as a predictor for therapeutic outcome in EC. Therefore, stratification of patients with type II EC based on their *PPP2R1A* mutational status, or of patients with type I EC based on their CIP2A or PME-1 status could help to establish more reliable testing of current and future targeted therapies in clinical trials. Furthermore, the presence of CIP2A or PME-1 expression could also broaden the therapeutic possibilities for the type I ECs. Dedicated pre-clinical studies in EC cells, with functional vs. dysfunctional PP2A status, should address these issues in the near future. Additionally, the presence of *PPP2R1A* mutations could also help to diagnose patients with type II EC in earlier stages (e.g., via liquid biopsies), thereby also contributing to a better patient outcome. However, further research is warranted in order to confirm this marker potential of *PPP2R1A* in type II ECs. In the end, only such dedicated studies, will help treatments for patients with type I and type II EC to catch-up with the emerging personalized medicine and targeted therapies already established in many other cancers.

## Author Contributions

MR: writing. VJ: review and Editing.

### Conflict of Interest Statement

The authors declare that the research was conducted in the absence of any commercial or financial relationships that could be construed as a potential conflict of interest.

## Abbreviations

Bad: Bcl2-associated death promotor
CIP2A: Cancerous inhibitor of PP2A
EC: Endometrial carcinoma
ERK: Extracellular signal-regulated kinase
GSK-3β: Glycogen synthase kinase 3β
HPV: Human papilloma virus
MAPK: Mitogen-activated protein kinase
MEK: Mitogen-activated protein kinase kinase
mTOR: Mammalian target of rapamycin
PI3K: Phosphatidylinositol-4,5-bisphosphate 3-kinase
PME-1: PP2A methylesterase 1
PP2A: Protein phosphatase 2A
pRb: Protein retinoblastoma
PTPA: Phosphatase 2A phosphatase activator
SET: Suvar/Enhancer of zeste/Trithorax
SMAP: Small molecule activator of PP2A
SV40: Simian virus 40
TKI: Tyrosine kinase inhibitor.
